# Blocking Complement Factor B Activation Reduces Renal Injury and Inflammation in a Rat Brain Death Model

**DOI:** 10.3389/fimmu.2019.02528

**Published:** 2019-11-01

**Authors:** Neeltina M. Jager, Judith E. van Zanden, Marta Subías, Henri G. D. Leuvenink, Mohamed R. Daha, Santiago Rodríguez de Córdoba, Felix Poppelaars, Marc A. Seelen

**Affiliations:** ^1^Department of Surgery, University Medical Center Groningen, University of Groningen, Groningen, Netherlands; ^2^Centro de Investigaciones Biológicas, Consejo Superior de Investigaciones Científicas, Madrid, Spain; ^3^Centro de Investigación Biomédica en Enfermedades Raras, Madrid, Spain; ^4^Division of Nephrology, Department of Internal Medicine, University Medical Center Groningen, University of Groningen, Groningen, Netherlands; ^5^Department of Nephrology, Leiden University Medical Center, Leiden, Netherlands

**Keywords:** factor B, complement, renal transplantation, brain death, donation

## Abstract

**Introduction:** The majority of kidneys used for transplantation are retrieved from brain-dead organ donors. In brain death, the irreversible loss of brain functions results in hemodynamic instability, hormonal changes and immunological activation. Recently, brain death has been shown to cause activation of the complement system, which is adversely associated with renal allograft outcome in recipients. Modulation of the complement system in the brain-dead donor might be a promising strategy to improve organ quality before transplantation. This study investigated the effect of an inhibitory antibody against complement factor B on brain death-induced renal inflammation and injury.

**Method:** Brain death was induced in male Fischer rats by inflating a balloon catheter in the epidural space. Anti-factor B (anti-FB) or saline was administered intravenously 20 min before the induction of brain death (*n* = 8/group). Sham-operated rats served as controls (*n* = 4). After 4 h of brain death, renal function, renal injury, and inflammation were assessed.

**Results:** Pretreatment with anti-FB resulted in significantly less systemic and local complement activation than in saline-treated rats after brain death. Moreover, anti-FB treatment preserved renal function, reflected by significantly reduced serum creatinine levels compared to saline-treated rats after 4 h of brain death. Furthermore, anti-FB significantly attenuated histological injury, as seen by reduced tubular injury scores, lower renal gene expression levels (>75%) and renal deposition of kidney injury marker-1. In addition, anti-FB treatment significantly prevented renal macrophage influx and reduced systemic IL-6 levels compared to saline-treated rats after brain death. Lastly, renal gene expression of IL-6, MCP-1, and VCAM-1 were significantly reduced in rats treated with anti-FB.

**Conclusion:** This study shows that donor pretreatment with anti-FB preserved renal function, reduced renal damage and inflammation prior to transplantation. Therefore, inhibition of factor B in organ donors might be a promising strategy to reduce brain death-induced renal injury and inflammation.

## Introduction

Although the field of renal transplantation has made huge progress over the last 50 years, one of the main challenges remains the disparity between demand and supply of renal allografts (1). Therefore, increasing efforts are made to expand, but also to optimize the current donor pool. Kidneys are retrieved from living donors, deceased after circulatory death (DCD) donors, and deceased after brain death (DBD) donors. Despite the increasing number of living donors, the majority of kidneys are still retrieved from DBD donors (1). However, brain death induces physiological disturbances characterized by hemodynamic changes, metabolic disturbances, and immunological derangements. Therefore, kidneys retrieved from DBD donors give inferior results, reflected by a higher rate of delayed graft function than their living counterparts (2).

An important denominator in brain death-induced renal inflammation is activation of the complement system (3, 4). The complement system can be activated by three different pathways: the classical pathway (CP), the lectin pathway (LP), and the alternative pathway (AP). Activation of each of these three pathways results in the cleavage of complement component C3 into C3a and C3b. Subsequently, activation of C3 leads to the formation of the C5 convertases, which cleave C5 into C5a and C5b. C5b is the initial protein for the formation of C5b-9, also known as the membrane attack complex (MAC). The MAC induces the formation of pores in the cell membrane, which results in cell lysis. Besides, C5b-9 induces tissue injury via intra-cellular pro-inflammatory signaling pathways (5). In addition, the anaphylatoxins C3a and C5a are produced, which provoke influx and activation of inflammatory cells (6, 7).

Early studies already have demonstrated the presence of complement C3 in kidneys from DBD rats (8). C3 deposition was seen on endothelial cells and in the glomeruli of DBD-derived kidneys, while no C3 deposition was observed in renal biopsies from living donors. In line with these results, C3d deposition was detected in renal biopsies from human DBD donors before reperfusion (9, 10), which suggest that C3d was deposited as a direct result of brain death itself. Complement C3 activation results in the production of the downstream activation products C5a and C5b-9, which are both systemically and locally upregulated in the DBD donor (11, 12). These studies demonstrate that the complement system is activated in DBD donors. Therefore, inhibition of the complement system might be an attractive strategy to attenuate brain death-induced renal injury (13).

A potential target for intervention in the DBD donor might be complement factor B. Factor B is one of the key components required for activation of the complement AP (9, 14). Bb, an active fragment of factor B, is significantly elevated in plasma from DBD donors compared to living donors (11). These plasma levels of Bb are positively correlated with systemic C5b-9 levels in DBD donors. High systemic C5b-9 levels in DBD donors are associated with a higher incidence of acute rejection in the recipient (11). In addition, local renal expression of factor B was significantly increased in kidneys from DBD donors compared to kidneys from living donors at baseline (15). These studies demonstrate that factor B is both systemically and locally upregulated in kidneys from DBD donors. Therefore, inhibition of factor B could be critical to protect against brain death-induced renal injury. To our knowledge, no studies have investigated whether inhibition of factor B is therapeutically effective in DBD donors.

Our study aimed to investigate whether inhibition of factor B can attenuate brain death-induced renal injury and inflammation. To do so, we pretreated rats with a monoclonal antibody against factor B (anti-FB) and subsequently subjected rats to 4 h of brain death. We found that pretreatment with anti-FB significantly improved renal function, reduced renal damage, and inflammation in brain-dead rats prior to transplantation.

## Materials and Methods

### Experimental Outline

**Figure 1:**
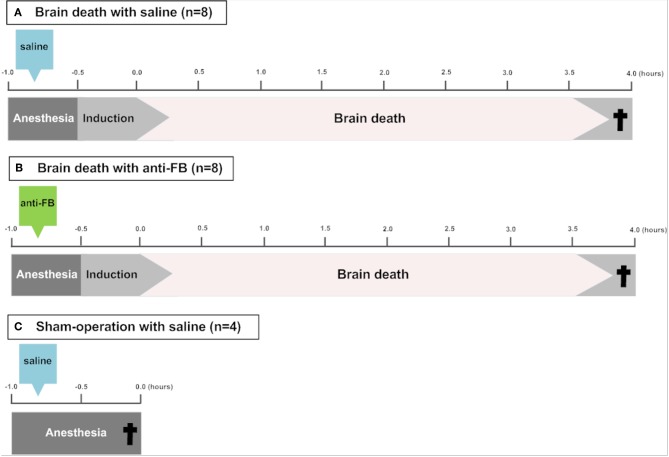
Schematic overview of the experimental set-up. To induce brain death, a catheter was placed in the epidural space through a frontal-lateral borehole in the skull and inflated slowly over 30 min. **(A)** Saline or **(B)** anti-factor B (anti-FB) was administered via the femoral vein 20 min before the induction of brain death. After 4 h of brain death, rats were sacrificed and blood, urine and kidneys were collected. In **(C)** sham-operated rats, a hole was drilled in the skull without insertion of the balloon catheter and rats were ventilated for 30 min under anesthesia before sacrifice. ^†^Termination. Anti-FB, anti-factor B.

In this study, 22 rats (two rats were excluded because of technical failures and replaced) were randomly divided into the following groups (Figure 1):

Brain death with saline (control group) (*n* = 8)
Brain death with anti-factor B (anti-FB) (*n* = 8)
Sham-operation with saline (*n* = 4).

### Rats

Adult male Fischer F344/NHsd rats (Envigo, Dublin, VA, USA) between 250 and 300 grams were used. Rats received food and water *ad libitum*. All experiments were performed at the local animal facility of the University Medical Center Groningen according to the Experiments on Animal Act (1996) issued by the Ministry of Public Health, Welfare and Sports of the Netherlands.

### Brain Death Procedure

To test whether inhibition of the complement AP protects against brain death-induced renal injury, we treated brain-dead rats with a mouse anti-human monoclonal antibody against factor B (FB28.4.2; IgG2b). FB28.4.2 (anti-FB) is produced in the laboratory of prof. Dr. Santiago Rodriquez de Cordoba at the Centro de Investigaciones Biológicas, Madrid, Spain. Anti-FB is an inhibitory antibody, which targets an evolutionary-conserved epitope in the Ba fragment of factor B. Anti-FB efficiently inhibits the formation of the AP C3 proconvertase in humans, rats and other species by blocking the interaction between the Ba domain of factor B and C3b (16). Twenty minutes before the start of brain death induction, 8 mg of purified anti-FB in a total volume of 750 μl, was administered via the femoral vein. Saline, used in the control group and sham-operated group, was also administered via the femoral vein at the same time and in the same volume as anti-FB. Brain death was induced as described previously (17). Briefly, rats were anesthetized, intubated and ventilated. Temperature and mean arterial pressure (MAP) were continuously monitored and regulated. A 4F Fogarty balloon catheter (Edwards Lifesciences LLC, Irvine, USA) was placed in the epidural space through a frontal-lateral borehole in the skull and inflated slowly. Brain death induction was completed after 30 min, and the apnoea test confirmed brain death. Subsequently, anesthesia was stopped and the ventilator was switched to a mix of 50% O_2_ and 50% medical air. A MAP above 80 mmHg was considered to be normotensive. When MAP dropped below 80 mmHg, 0.1 kg/L hydroxyethyl starch solution (HAES-steril 10%, Fresenius Kabi, Bad Homburg, Germany) and, if necessary, 0.01 mg/ml norepinephrine were administered. A maximum of 800 μl of fluid was administered during 4 h of brain death. Fluid administration was comparable in all groups (Table 1). Blood, urine and kidneys were harvested after 4 h of brain death. Kidneys were embedded in paraffin or snap-frozen in liquid nitrogen and together with the serum and urine stored at −80°C. Sham-operated rats underwent the same surgical procedure, but without the insertion of the balloon catheter, and only received saline. Sham-operated rats were ventilated for 30 min under anesthesia with a mixture of 2.5% isoflurane and 100% O_2_. After 30 min, sham-operated rats were sacrificed.

**Table 1:** Overview of the fluids administered, when mean arterial pressure dropped below 80 mmHg, in rats during the 4 h brain death period.

**Group**	**Brain death + saline**		**Brain death + anti-factor B**		***p*-value**
	**Median**	**IQR**	**Median**	**IQR**	
HAES (ml)	1	0–1.5	0.8	0–1.5	0.479
Noradrenaline (ml)	0.295	0–1.9	0.25	0–2.88	0.989
MAP (mmHg)	100	90–110	103	79–115	0.787

*IQR, interquartile range; HAES, hydroxyethyl starch solution; MAP, mean arterial pressure*.

### Renal Function

To investigate renal function, serum creatinine levels were measured at the time of sacrifice, using a Roche Modular P system (Roche, Basel, Switzerland).

### Renal Morphology

Paraffin sections (4 μm) were stained with Periodic Acid-Schiff stain. Histological injury, reflected by acute tubules necrosis (ATN) was determined semi-quantitatively by two observers using the following scoring system: 0 = no ATN; 1 = 0–10%; 2 = 10–25%; 3 = 25–50% and 4 = ≥50% ATN.

### Immunohistochemistry

Immunohistochemical stainings for C3d, C5b-9, and neutrophils (HIS48) were performed on frozen sections (4 μm) fixed in acetone. Immunohistochemistry for kidney injury molecule-1 (KIM-1) and macrophages (ED-1) were performed on formalin-fixed, paraffin-embedded sections. Paraffin sections (4 μm) were deparaffinized and rehydrated. 0.1M Tris/HCl (pH 9) was used as an antigen retrieval buffer. All sections were blocked with hydrogen peroxidase for 30 min and incubated with the primary antibody for 1 h at room temperature (Table 2). After washing with phosphate buffered saline (PBS), the slides were incubated with the appropriate horseradish peroxidase-conjugated secondary and tertiary antibodies (Dako, Carpinteria, USA) in 1% BSA solution for 30 min. The reaction was developed by addition of 3-amino-9-ethylcarbazole (AEC; Dako) or 3,3-diaminobenzidine (DAB; Merck, Darmstadt, Germany) and 0.03% H_2_O_2_. Sections were counterstained with hematoxylin and embedded in Aquatex mounting agent (Merck).

**Table 2:** Primary antibodies used for immunohistochemistry.

**Antibody**	**Sections**	**Host and target species**	**Supplier**	**2nd/3rd antibodies**
C3d	Frozen	Rabbit polyclonal anti-human C3d	A0063, Dako, Carpinteria, USA	GαRb^PO^/RbαG^PO^
C5b-9	Frozen	Mouse monoclonal anti-rat membrane attack complex	Hycult, Uden, the Netherlands	RbαM^PO^
ED-1	Paraffin	Mouse monoclonal anti-rat macrophages/monocytes	Abcam, Oxford, UK	RbαM^PO^/GαRb^PO^
HIS48	Frozen	Mouse monoclonal anti-rat granulocytes	IQProducts, Groningen, the Netherlands	RbαM^PO^/GαRb^PO^
KIM-1	Paraffin	Mouse monoclonal anti-human KIM-1 AKG7	Biogen Inc, Cambridge, Massachusetts, USA	RbαM^PO^/GαRb^PO^

*G, goat; Rb, rabbit; M, mouse; PO, polyclonal; KIM-1, kidney injury molecule-1*.

For quantitative evaluation of HIS48 and ED-1 in the renal cortex, 40 snapshots of the renal cortex were taken from the representative sections using ImageJ Software (National Institutes of Health). Cells positive for each marker were then counted using Aperio ImageScope Analysis Software (objective 50x−200x) (Leica Biosystems, Vista, CA, USA). C3d, C5b-9, and KIM-1 sections were scored semi-quantitatively by two observers at a magnification of 20x. C3d and C5b-9 were scored by the following scoring system: 0 = no staining; 1 = 0–25%; 2 = 25–50%; 3 = 50–75% and 4 = 75–100% staining in the renal cortex. KIM-1 was scored by estimating the percentage of KIM-1 expression in the cortical tubules. KIM-1 was scored per field, as described by van Timmeren et al. (18). In brief, 0 = no staining; 1/2 = 0–12.5%; 1 = 12.5–25%; 2 = 25–50%; 3 = 50–75% and 4 = 75–100%.

### IL-6 ELISA

Plasma protein levels of IL-6 were determined by a rat enzyme-linked immunosorbent assay (R&D Systems; DY506, Abingdon, Oxon, UK). All samples were analyzed in duplicate and measured at an optical density of 450 nm.

### C3d ELISA

Rat C3d ELISA was performed, as described previously, to measure complement activation after 4 h of brain death (19). In brief, rat C3d was captured with a monoclonal mouse anti-C3 antibody (sc-28294, Santi Cruz, CA, USA). A rabbit anti-human C3d was used as detection antibody (Dako) and goat anti-rabbit-HRP (Dako) with 3,3′,5,5′-Tetramethylbenzidine (TMB) K-Blue as substrate. Sample incubation and detection steps were performed for 60 min at 37°C. Before C3d was measured, all samples were polyethylene glycol (PEG) precipitated. PEG precipitation is required since free C3d shares epitopes with intact C3. All plasma samples were 1:1 diluted with 22% PEG in 0.1M borate/EDTA buffer (pH 8.32). Samples were incubated for 3 h on ice and subsequently centrifuged for 10 min at 4000 rpm at 4°C. Then, supernatants were collected and used for C3d quantification. A standard curve was made using zymosan-activated pooled rat plasma. The amount of C3d in the samples was determined from the standard curve and expressed in arbitrary units/ml. Samples were analyzed in duplicate and measured at an OD of 450 nm (VICTOR-3, 1420 multilabel counter, PerkinElmer, Waltham, US).

### RNA Isolation

RT-qPCR was performed to investigate the renal gene expression levels of pro-inflammatory cytokines after brain death. Total RNA was extracted from frozen kidneys using the TRIzol method (Invitrogen, Waltham, US) and DNase Amplification Grade (Merck), according to manufacturer's instructions. Genomic DNA contamination was verified by RT-qPCR using β-actin primers, in which the addition of reverse transcriptase was omitted.

### cDNA Synthesis and qPCR

cDNA synthesis was performed by the addition of 0.5 μl sterile water, 4 μl 5x first strand buffer (Invitrogen), 2 μl DTT (Invitrogen), and 1 μl M-MLV Reverse Transcriptase (Invitrogen) and primers (Table 3). The mixture was then incubated for 50 min at 37°C. After that, the reverse transcriptase was inactivated by incubating the mixture at 70°C for 15 min. The Taqman Applied Biosystems 7900HT RT-qPCR system (Biosystems, Carlsbad, USA) was used to amplify and detect PCR products, using SYBR Green (Applied Biosystems, Foster City, USA). Ct values were corrected for ß-actin and gene expression values were expressed as 2^−ΔΔ*CT*^ (Ct: threshold cycle).

**Table 3:** Gene-specific qPCR primers.

**Primers**	**Primer sequences**	**Amplification length**
β-actin	5′-GGAAATCGTGCGTGACATTAAA-3′	74
	5′-GCGGCAGTGGCCATCTC-3′	
BAX	5′-GCGTGGTTGCCCTCTTCTAC-3′	74
	5′-TGATCAGCTCGGGCACTTTAGT-3′	
Bcl-2	5′-CTGGGATGCCTTTGTGGAA-3′	70
	5′-TCAGAGACAGCCAGGAGAAATCA-3′	
IL-1β	5′-CAGCAATGGTCGGGACATAGTT-3′	106
	3'-GCATTAGGAATAGTGCAGCCATCT-5′	
IL-6	5′-CCAACTTCCAATGCTCTCCTAATG-3′	89
	5′-TTCAAGTGCTTTCAAGAGTTGGAT-3′	
IL-18	5′-CAACCGCAGTAATACGGAGCATA-3′	62
	5′-CAGGCGGGTTTCTTTTGTCA-3′	
KIM-1	5′-AGAGAGAGCAGGACACAGGCTT-3′	75
	5′-ACCCGTGGTAGTCCCAAACA-3′	
MCP-1	5′-CTTTGAATGTGAACTTGACCCATAA-3′	78
	5′-ACAGAAGTGCTTGAGGTGGTTGT-3′	
P-selectin	5′-TCTCTGGGTCTTCGTGTTTCTTATCT-3′	80
	5′-GTGTCCCCCTAGTACCATCTGAA-3′	
VCAM-1	5′-TGTGGAAGTGTGCCCGAAA-3′	84
	5′-ACGAGCCATTAACAGACTTTAGCA-3′	

*β-actin, βeta-actin; BAX, Bcl-2-associated X protein; Bcl-2, B-cell lymphoma protein 2; IL, Interleukin; KIM-1, kidney injury molecule-1; MCP-1, monocyte chemoattractant protein-1; VCAM-1, vascular cell adhesion molecule-1*.

### Statistical Analysis

Statistical analyses were performed with IBM SPSS Statistics 23 (IBM Corp, Armonk, NY, USA). The Kruskal-Wallis test was performed for multiple group comparisons. The Mann-Whitney U test was used to compare the differences between two groups. Bonferroni correction was used to account for multiple comparisons. All statistical tests were 2-tailed and a *p* < 0.05 was considered significant. Non-parametric data are presented as median ± interquartile range and parametric data are displayed as mean ± SD.

## Results

### Treatment With Anti-factor B Prevents Both Systemic and Local Complement Activation in Rats Subjected to Brain Death

**Figure 2:**
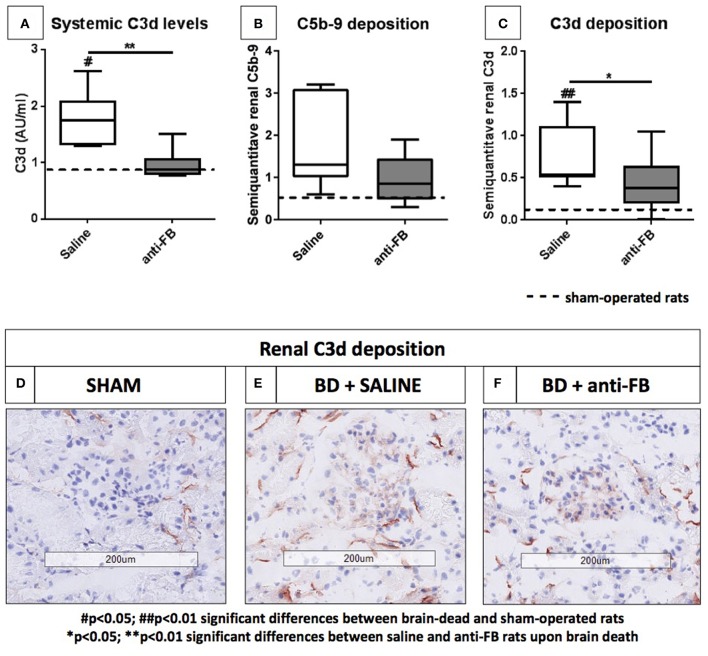
Systemic and local complement levels after 4 h of brain death. **(A)** Systemic C3d levels of brain-dead rats treated with saline or anti-factor B. Plasma C3d levels were determined after 4 h of brain death. C3d was captured by using a monoclonal mouse anti-C3 antibody, detected with a rabbit anti-human C3d antibody and goat anti-rabbit-HRP. **(B)** Renal C5b-9 deposition and **(C)** renal C3d deposition in frozen sections from **(D)** sham-operated rats, **(E)** saline-treated rats, and **(F)** anti-factor B treated rats after 4 h of brain death. Data are shown as median ± IQR. Data were analyzed by Mann Whitney-*U*-test and Bonferroni *post-hoc* test, asterisks above the bars denote significant differences between the brain-dead rats (*p < 0.05, **p < 0.01, and ***p < 0.001). The dashed line represents the mean of the sham-operated rats. ^#^Significant differences between the brain-dead rats vs. sham-operated rats (^#^*p* < 0.05, ^##^*p* < 0.01, and ^###^*p* < 0.001). Anti-FB, anti-factor B.

To investigate whether the complement system is activated in our rat brain death model, we determined systemic and local complement activation levels after 4 h of brain death. Systemic C3d levels were significantly increased after the induction of brain death (Figure 2A, *p* < 0.05) when compared to sham-operated rats, which indicates that the complement system was indeed activated upon brain death.

Next, we assessed whether treatment with anti-FB was able to prevent systemic complement activation in rats. Pretreatment with anti-FB prevented complement activation significantly, shown by comparable C3d levels as found in sham-operated rats (Figure 2A, *p* < 0.01). In addition, we determined whether treatment with anti-FB led to less local complement activation. There was no significant increase in C5b-9 deposition after 4 h of brain death compared to sham-operated rats (Figure 2B). However, renal C3d deposition was significantly increased in brain-dead rats compared to sham-operated rats (Figure 2C, *p* < 0.01). In addition, brain-dead rats pretreated with anti-FB had significantly less renal C3d deposition than saline-treated rats (Figures 2C–F, *p* < 0.05). Overall, anti-FB significantly prevented both systemic and local complement activation on the level of C3 after 4 h of brain death.

### Anti-factor B Preserves Renal Function and Attenuates Renal Injury After Brain Death

**Figure 3:**
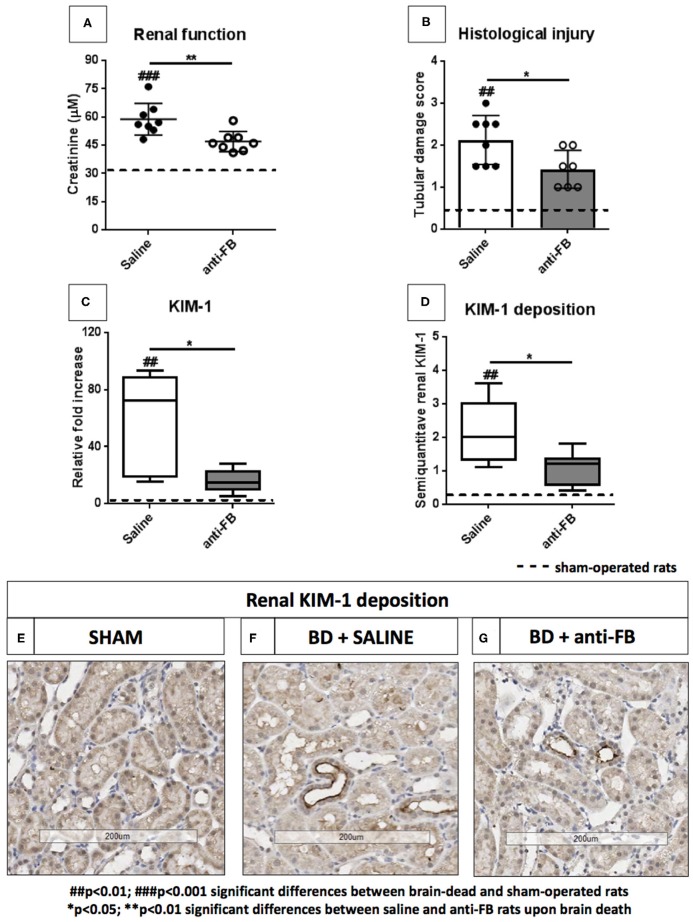
Renal function and renal injury upon brain death with saline or anti-factor B. **(A)** Renal function reflected by plasma creatinine levels of brain-dead rats treated with saline or anti-FB compared to sham-operated rats. Serum creatinine levels were measured at time of sacrifice, using a Roche Modular P system. **(B)** Tubular damage was reflected as a percentage of acute tubular necrosis in the renal cortex using a semi-quantitative method. **(C)** Renal KIM-1 gene expression in brain-dead and sham-operated rats treated with saline or anti-FB was determined by quantitative real-time PCR. The mRNA expression of KIM-1 relative to β-actin was set at 1 in sham-operated rats, the other values, are calculated accordingly. **(D)** Renal KIM-1 deposition after 4h of brain death, KIM-1 was scored by estimating the percentage of KIM-1 expression in the cortical tubules. **(E)** KIM-1 deposition in sham-operated rats, **(F)** in saline-treated rats, and **(G)** anti-FB treated rats. Data are shown as median ± IQR. Data were analyzed by Mann Whitney-*U*-test and Bonferroni *post-hoc* test, asterisks above the bars denote significant differences between the brain-dead rats (*p < 0.05, **p < 0.01, and ***p < 0.001). The dashed line represents the mean of the sham-operated rats. ^#^Significant differences between the brain-dead rats vs. sham-operated rats (^#^*p* < 0.05, ^##^*p* < 0.01, and ^###^*p* < 0.001). Anti-FB, anti-factor B.

To determine whether treatment with anti-FB was able to preserve renal function and protect against renal injury, we measured serum creatinine levels, scored for histological injury and investigated kidney injury molecule-1 (KIM-1) levels in the kidney. First, plasma creatinine levels were significantly elevated after brain death compared to sham-operated rats (Figure 3A, *p* < 0.001). Pretreatment with anti-FB preserved renal function, reflected by significantly lower serum creatinine levels than saline-treated rats after brain death (Figure 3A, *p* < 0.01). However, serum creatinine levels in anti-FB treated rats were still significantly higher than in sham-operated rats. Second, anti-FB treated rats had significant less renal injury than saline-treated brain-dead rats, demonstrated by lower levels of renal tubular necrosis (Figure 3B, *p* < 0.05). Third, mRNA expression of KIM-1, a protein which is mainly expressed on damaged renal epithelial cells (18), was significantly upregulated in brain-dead rats compared to sham-operated rats. Pretreatment with anti-FB resulted in significantly lower KIM-1 gene expression levels, which indicates a reduction in renal tubular damage (Figure 3C, *p* < 0.05). Lastly, we analyzed renal KIM-1 deposition by performing immunohistochemistry. Brain-dead rats had significant more KIM-1 protein deposition in the proximal renal tubules than sham-operated rats. After brain death, KIM-1 staining was primarily seen in the corticomedullary junction. Anti-FB treated rats had significantly less KIM-1 deposition than saline-treated rats (Figures 3D–G, *p* < 0.05). Taken together, these results show that pretreatment with anti-FB preserved renal function and attenuated brain death-induced renal injury.

### Anti-factor B Reduces Systemic IL-6 Levels and Expression Levels of Pro-inflammatory Genes in the Kidney

**Figure 4:**
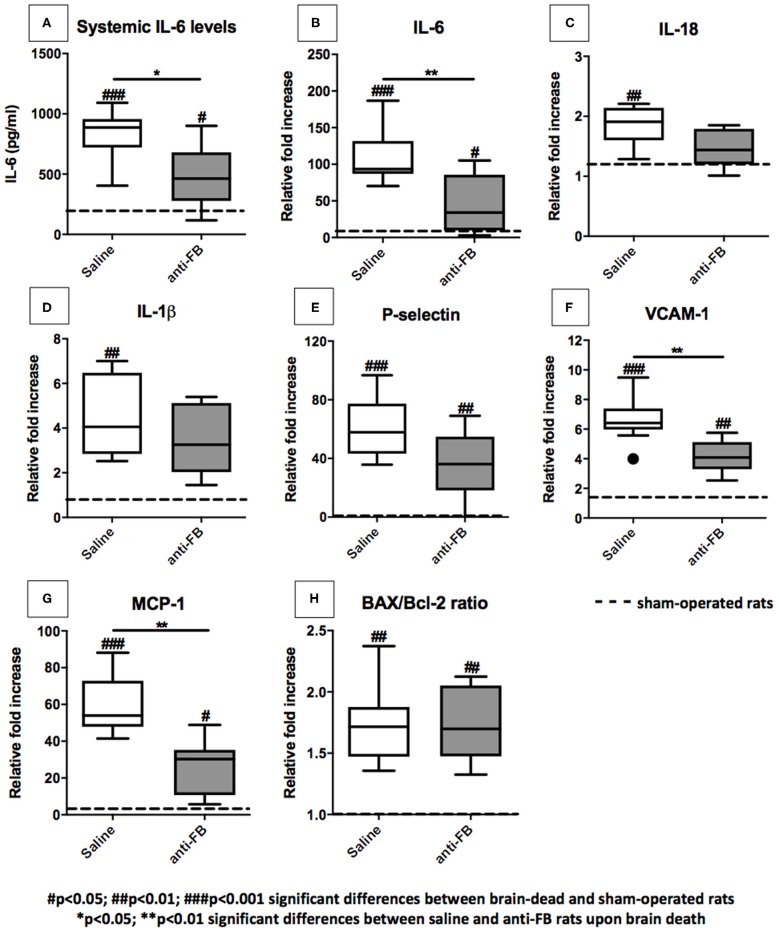
Pro-inflammatory gene expression levels in the kidney after 4 h of brain death. **(A)** Systemic IL-6 levels of brain-dead rats treated with saline or anti-FB compared to sham-operated rats after 4 h of brain death. A rat enzyme-linked immunosorbent assay determined plasma levels of IL-6. All samples were analyzed in duplicate and measured at an OD of 450 nm. Pro-inflammatory gene expressions in the kidneys of brain-dead rats treated with saline or anti-factor B. mRNA expressions of **(B)** IL-6, **(C)** IL-18, **(D)** IL-1β, **(E)** P-selectin, **(F)** VCAM-1, **(G)** MCP-1, and **(H)** BAX/Bcl-2 ratio. Data are shown as expression relative to β-actin as set at 1 in sham-operated rats, the other values, are calculated accordingly. Data are shown as median ± IQR. Data were analyzed by Mann Whitney-*U*-test and Bonferroni *post-hoc* test, asterisks above the bars denote significant differences between the brain-dead rats (*p < 0.05, **p < 0.01, and ***p < 0.001). The dashed line represents the sham-operated rats. ^#^Significant differences between the brain-dead rats vs. sham-operated rats (^#^*p* < 0.05, ^##^*p* < 0.01, and ^###^*p* < 0.001). Anti-FB, anti-factor B.

To assess whether complement inhibition with anti-FB influences brain death-induced renal inflammation, we first determined systemic IL-6 levels after 4 h of brain death. IL-6 plasma levels were significantly higher in saline-treated brain-dead rats than in sham-operated rats (Figure 4A, *p* < 0.001). Pretreatment with anti-FB prevented the increase of systemic IL-6 significantly compared to saline-treated rats after brain death (Figure 4A, *p* < 0.05). Next, we determined renal mRNA levels of multiple pro-inflammatory genes. After 4 h of brain death, the pro-inflammatory cytokines IL-6, IL-18, and IL-1β, adhesion molecules P-selectin and VCAM-1, chemokine MCP-1, and apoptosis ratio BAX/Bcl-2 were all significantly upregulated compared to sham-operated rats (Figures 4B–H). These data demonstrate that our rat brain death model mimics the injury seen in human brain-dead donors (20). Rats treated with anti-FB showed significantly lower renal gene expression levels of pro-inflammatory cytokine IL-6 (Figures 4B,C, *p* < 0.01). Besides, renal gene expression levels of VCAM-1 and MCP-1 were significantly lower in anti-FB treated rats than in saline-treated rats upon brain death (Figures 4F,G, *p* < 0.01). Gene expression levels of IL-1β, IL-18, and P-selectin were lower as well, but not significantly (Figures 4F,G). Treatment with anti-FB did not reduce the apoptosis ratio BAX/Bcl-2 in the kidney upon brain death (Figure 4H). Altogether, the results demonstrate that anti-FB attenuates the pro-inflammatory response upon brain death.

### Treatment With Anti-factor B Reduces the Influx of Macrophages After 4 h of Brain Death

**Figure 5:**
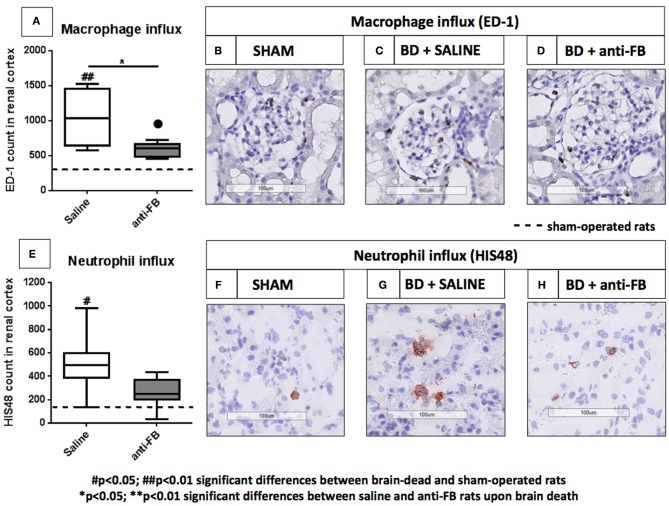
Infiltrated leukocytes in the kidney after brain death in saline or anti-factor B treated rats. Infiltrated leukocytes in kidneys of sham-operated and brain-dead rats treated with saline or anti-factor B. **(A)** Renal neutrophil influx was scored in frozen sections from **(B)** sham-operated rats, **(C)** brain-dead rats treated with saline, or **(D)** brain-dead rats treated with anti-factor B. **(E)** Renal macrophage influx was scored in paraffin-embedded sections from **(F)** sham-operated rats, **(G)** brain-dead rats treated with saline, or **(H)** anti-factor B. Sections were quantified for the number of positive cells in the renal cortex using Aperio Imagescope (objective 50x−200x) and ImageJ Software. All pictures are presented at 200x magnification. Data are shown as median ± IQR. Data were analyzed by Mann Whitney-*U*-test and Bonferroni *post-hoc* test, asterisks above the bars denote significant differences between the brain-dead rats (*p < 0.05, **p < 0.01, and ***p < 0.001). The dashed line represents the sham-operated rats. ^#^Significant differences between the brain-dead rats vs. sham-operated rats (^#^*p* < 0.05, ^##^*p* < 0.01, and ^###^*p* < 0.001). Anti-FB, anti-factor B.

To complete the renal inflammatory profile, we determined the influx of neutrophils and macrophages in the kidney by immunohistochemistry. After 4 h of brain death, the number of macrophages (ED-1) and neutrophils HIS(48) in the kidney were significantly increased in brain-dead rats compared to sham-operated rats. Next, we investigated the effect of anti-FB on the influx of leukocytes in the kidney. In brain-dead rats, treatment with anti-FB led to a significantly lower number of macrophages in the kidney than in saline-treated rats (Figures 5A–D, *p* < 0.01). In addition, the absolute number of neutrophils in the kidney was lower in anti-FB treated rats than in saline-treated rats (Figures 5E–H). In conclusion, pretreatment with anti-FB seems to attenuate the influx of leukocytes, especially macrophages, in the kidney upon brain death.

## Discussion

In this study, we investigated whether inhibition of factor B can attenuate brain death-induced renal injury and inflammation in rats. To achieve this, we pretreated rats with a monoclonal antibody against factor B (anti-FB) and subsequently subjected the rats to 4 h of brain death. We found that anti-FB preserved renal function, reduced renal injury and renal inflammation in brain-dead rats.

First, we studied the effect of anti-FB treatment on systemic and local complement activation. We observed significantly lower plasma levels of C3d in anti-FB treated rats than in saline-treated rats after 4 h of brain death. These results demonstrate that treatment with anti-FB prevents systemic complement activation, which might be important because high systemic complement levels in the donor are associated with acute rejection of human renal allografts (11). In addition, the present study demonstrates that pretreatment with anti-FB resulted in significant less local complement C3d deposition. Of importance, since previous studies have shown that renal complement C3 synthesis is associated with acute renal transplant rejection and acute post-ischemic renal failure (3, 10, 21, 22).

Next, we investigated the effect of treatment with anti-FB on renal function and renal injury after 4 h of brain death. Treatment with anti-FB preserved renal function in brain-dead rats. However, serum creatinine levels in anti-FB treated rats were still higher than creatinine levels measured in sham-operated rats. We ascribe these small differences in serum creatinine levels to the hemodynamic changes seen during brain death, which were not seen in sham-operated rats. Therefore, rats subjected to brain death received more fluid than sham-operated rats, which explains the observed differences in plasma creatinine levels between anti-FB treated rats and sham-operated rats (Table 1). In addition, we observed an increase in KIM-1 gene expression levels and KIM-1 deposition in the renal cortex of brain-dead rats compared to sham-operated rats. Similar observations were done by Nijboer et al., who showed that KIM-1 is substantially upregulated in human brain-dead donors (23). The fact that both KIM-1 gene expression levels and KIM-1 deposition were reduced in anti-FB treated rats seems to be important since KIM-1 is known to be an independent predictive marker for renal function in recipients after renal transplantation (24). Lastly, since we observed that treatment with anti-FB led to lower gene expression levels of IL-6, VCAM-1, and MCP-1 after brain death, it is suggested that anti-FB reduces brain death-induced renal inflammation. Interestingly, rats treated with anti-FB had systemic IL-6 levels comparable to sham-operated rats. These low IL-6 levels could be explained by the fact that Ba and Bb fragments have a variety of biological activities independent of the proteolytic activity (25–27).

An important limitation of this study is the fact that anti-FB was not administered after the confirmation of brain death. Anti-FB was administered 20 min before the start of brain death induction, which is impossible to realize in clinical practice (28). Therefore, this study set-up serves as a proof of principle to investigate the effect of anti-FB on brain death-induced renal injury in rats. More research is needed to evaluate the effect of anti-FB during different time points throughout the brain death period. In the current study, we did not include a group with anti-FB administration after the induction of brain death. The optimal time point of intervention would be between 30 and 90 min after brain death induction, which leaves only 2–3 h to evaluate the effect of anti-FB treatment on renal injury in our brain death model for rats. We consider this too short, since the maximum effect of anti-FB treatment is apparent after 4 h (16). Therefore, we consider it as a next step to investigate the effect of anti-FB treatment after the induction of brain death in a larger animal model for brain death. Another limitation of this study is that we only used male rats, to circumvent sex-related differences in complement levels and functionality (14, 29).

A study performed by Thurman et al. already demonstrated the potential of complement factor B inhibition in a mouse model of renal ischemia/reperfusion. An inhibitory monoclonal antibody to mouse factor B was used, which significantly preserved renal function and led to less renal injury (30, 31). Thus, factor B seems to be a promising target to improve renal transplant outcome, in both the donor as well as in the recipient. However, treatment of the donor might be more beneficial than of the recipient, since the complement system is already activated in the donor and as has been shown to affect the function of the renal allograft (11, 32). Taken together, these results create a new window of opportunity for complement-targeted therapies in the renal transplantation setting.

However, when treating the donor, it should be considered that all organs will be treated with the same drug and same dose of treatment. While our study shows that the quality of the kidney improves from treatment with anti-FB in the donor, this is not yet investigated for the other organs. Based on literature, the heart may also benefit from treatment with anti-FB. Chun et al. showed that systemic levels of factor B in both mice and human are positively correlated with myocardial necrosis after cardiac ischemia/reperfusion injury. In addition, absence of factor B resulted in significant myocardial protection after cardiac ischemia/reperfusion (33). Although less is known about factor B in other organs during transplantation, factor B has been described to play a pivotal role in multiple pro-inflammatory disease models, such as retinal injury and arthritis (34, 35). Altogether, these positive findings resulted in the development of therapeutic targets against factor B.

Currently, two drugs that can inhibit factor B are tested in clinical trials. One of them is LPN023, a small molecule that binds the active site of factor B. LPN023 is currently tested in phase II dose-ranging study in IgA nephropathy patients [NCT03373461; (36)]. Recently, Ionis Pharmaceuticals announced to start a phase II study with their antisense drug against factor B in patients with age-related macular disease (37). Various agents that target factor B are currently under development, for example siRNA against factor B [Alnylam; (38)]. These trials, may soon lead to the clinical availability of one or more complement inhibitors that target factor B.

In conclusion, we show that anti-FB pretreatment in brain-dead donor rats preserves renal function and protects against renal injury and renal inflammation. Therefore, anti-FB treatment might be a potential therapy to reduce brain death-induced renal injury prior to transplantation.

## Data Availability Statement

The datasets generated for this study are available on request to the corresponding author.

## Ethics Statement

The animal study was reviewed and approved by the Animal Welfare Body of the Institutional Animal Care and Use Committee at the University Medical Center Groningen, University of Groningen, Groningen, The Netherlands.

## Author Contributions

Research idea and study design by NJ, JZ, MS, MD, SR, FP, and MAS. NJ and JZ planned and conducted the experiments and wrote the manuscript. Data analysis and interpretation of the results by NJ, JZ, HL, MD, FP, and MAS. Statistical analysis by NJ and JZ. All authors were involved in editing the final manuscript. All authors read and approved the final manuscript.

### Conflict of Interest

The authors declare that the research was conducted in the absence of any commercial or financial relationships that could be construed as a potential conflict of interest.

## Abbreviations

AEC: 3-amino-9-ethylcarbazole
Anti-FB: anti-factor B
AP: alternative pathway
ATN: acute tubular necrosis
CP: classical pathway
DAB, 3: 3-diaminobenzidine
DBD: donation after brain death
DCD: donation after circulatory death
HAES: hydroxyethyl starch solution
KIM-1: kidney injury molecule-1
LP: lectin pathway
MAC: membrane attack complex
MAP: mean arterial pressure
PBS: phosphate buffered saline
PEG: polyethylene glycol
TMB, 3,3′ ,5: 5′-tetramethylbenzidine.
